# Resident’s Perspective on Developing Community-Based Tourism – A Qualitative Study of Muen Ngoen Kong Community, Chiang Mai, Thailand

**DOI:** 10.3389/fpsyg.2020.01493

**Published:** 2020-07-24

**Authors:** Yu-Chih Lo, Pidpong Janta

**Affiliations:** ^1^Department of Leisure Industry Management, National Chin-Yi University of Technology, Taichung, Taiwan; ^2^Department of Business Administration, National Chin-Yi University of Technology, Taichung, Taiwan

**Keywords:** community-based tourism, benefits and challenges, community development, Chiang Mai, thematic analysis, case study

## Abstract

Community-Based Tourism (CBT) has been presented as an alternative to sustaining tourism development in developing countries. This tourism model offers local residents an opportunity to manage natural and cultural resources in order to promote the local economy and generate greater benefits. The objective of the study is to investigate the benefits and challenges of CBT as well as solutions to address identified shortcomings by studying Muen Ngoen Kong community in Chiang Mai, Thailand. In order to achieve these objectives, qualitative methods, field observations, and interviews were employed, and qualitative data were analyzed using thematic analysis. The results of the field observation and interview data from local residents were reported, analyzed, and discussed. To practice CBT, the findings indicated that several challenges had been experienced in the implementation of CBT, including conflict over resource ownership and benefit leaking, financial issues, and problems of community participation. However, an abundance of tourism resources and security related concerns were identified as benefits of CBT in the area. In close collaboration with government agencies, product development was recommended to create a unique condition for CBT and address the shortcomings. It is crucial to involve local residents, empower the local community, conserve and cultivate cultural resources, and, finally, to maintain the overall sustainability of tourism resources.

## Introduction

### Changes in Tourist Behaviors

In recent decades, tourist behavior has begun to change as tourists seek a new inexperienced approach to destinations, where they can experience local culture and involve themselves in their travels. Therefore, their intent to travel is not only to explore new existing destinations, but also culture, ethics, ancient indigenous remains, and local history in which they can go beyond ordinary travels and have authentic and meaningful experiences based on personal perceptions. Likewise, from a tourism perspective, as tourism has become an activity to serve travel purposes and boost a country’s economy, it also allows local communities to offer an opportunity for the tourists to learn and enjoy their culture, promoting their own heritage and historical story (López-Guzmán et al., 2011). Based on the basis of initiatives and the management of natural and cultural resources, the local community must be the one who serves tourists a tourism product; however, by doing that, it also brings limits of tourism improvement to the local community. Considering these aspects, the local community has increasingly been recognized as important in tourism development and in the future direction of tourism (Butler and Pearce, 2003; Mason, 2003; Telfer and Sharpley, 2007).

Since the 1980s, tourism literature has observed that a basic key resource in tourist sustainability is involvement and inclusion of local residents in local communities (Hardy et al., 2002). Community participation is considered as one of the most necessary tools for rural community development. Woodley (1993) ensures that community participation creates sustainability and better opportunities for local residents by generating valuable benefits from tourism in local residents’ locality. Tosun (2006) added that tourism promotes the conservation of local resources and offers employment opportunities, tourism revenue, and infrastructure improvement (Liu and Var, 1986; Mehta and Kellert, 1998; Archer et al., 2001; Lindberg, 2001). Participation at a local level is primarily required to develop tourism planning and secure community economic growth (Murphy, 1985), which leads to local economic development, by influencing business, industries, and job opportunities in communities (Roseland and Connelly, 2005). To promote community participation, the concept of sustainable development has been called for improvement of life quality in the communities. This concept emphasizes and relies on issues of social quality and environmental responsibility. For this reason, the development gives residents with different levels of income and skills opportunities and provides them with a better quality of life, and, more importantly, provides their locality with environmental protection (Roseland and Connelly, 2005). Local participation encourages community empowerment and involvement in decision-making, as well as identification of local problems and difficulties (France, 2003). Lea (1988) points out that tourism will make local residents feel less valued if they are not empowered and fully participate in tourism developments. Consequently, sustainable outcome from tourism will be less likely to be generated (Lea, 1988). Perhaps, local resident’s potential and local knowledge increases the possibility that community involvement can be of major importance in tourism development. Essentially, the participation of the local community is important in this industry as it ensures that tourists get a memorable, unforgettable visiting experience and enables the community to gain benefits from the their visits at the same time. Residents offer helpful supporting data in decision-making processes due to their local knowledge; therefore, tourism planning and development must utilize resident’s potential and encourage local involvement. Opportunities to access markets and grow businesses in communities can be created by doing so, thus offering job opportunities and poverty reduction in rural areas.

### Tourist Behavior in Thailand

The context for this study is Thailand, which is known as the second largest economy in Southeast Asia. Its high ratio of revenue is in relation to the informal economy (Çakmak et al., 2018). Thailand has witnessed an increasing number of tourists in recent years. With over 73 million visitors in 2017 (Tourism Statistics 2017”, 2017), Thailand has become a popular travel destination for tourists all over the world. Chinese tourists in particular are attracted to the country, especially after the launch of the movie “Lost in Thailand” which was filmed in many cities in Thailand. In this sense, Chinese tourists have become and are considered as the largest group of tourists visiting Thailand. According to Tourism Statistic in 2019 (Domestic Tourism Statistics (Classify by region and province 2019), 2019), there were 1.1 million tourists who visited Chiang Mai in January, in which 28.5% of them were foreign tourists. The number of tourists visiting Chiang Mai has increased since last year by 1.11% (Domestic Tourism Statistics (Classify by region and province 2019), 2019). With the increasing number of tourists over recent years, Chiang Mai, once unpopular among tourists, has been rapidly explored by new groups of visitors. Chiang Mai, literally described as “a new city in the former time,” is the second largest city in Thailand. The city has the busiest international airport in Thailand’s northern part and contains a variety of natural resources, unique cultural heritage sites, and popular adventurer destinations, which attract international tourists. This city without a doubt attracts migrants from surrounding rural areas and neighboring countries. This phenomenon, both in number and choice of natural and cultural destinations, increasingly suggests a new trend in tourist behavior in Thailand.

The sections below are organized as follows. Section “Literature Review” introduces the concept of community tourism as an alternative means of tourism in developing countries, as well as in Thailand, and its related previous studies. Section “Methodology” presents the research design, data collection, and methods, interviewees’ profiles, and description of the research site. Section “Results” provides the findings of key concepts and themes derived from interviews. Sections “Discussion,” “Conclusion,” and “Suggestion for Future Studies” include explanations of the research site’s current circumstance, benefits, and challenges toward tourism development from both the community committee and local perspectives, and suggestions for future research.

## Literature Review

### Community-Based Tourism

Community-Based Tourism (CBT) has been presented as an alternative means to traditional mass tourism in developing countries, and is developed as a community development tool that aids communities in taking control over tourism management and development and deliver benefits to the communities which are generated by tourism activities (Trejos and Chiang, 2009). Additionally, CBT as a community development tool helps to strengthen and empower remote communities by assisting in tourism resource management and ensuring community participation (Jamal and Getz, 1995; Travel, 2009).

### Concept of CBT

The concept of CBT was first presented in Murphy’s work (Murphy, 1985) in which it is presented as directly related to community tourism in developing countries and was developed further to a greater extent by Murphy in 2004 (Murphy and Murphy, 2004). The concept, in accompaniment with other existing works, advanced research and opportunities for tourism development in rural areas. There are other existing models of tourism, including Pro-Poor Tourism (PPT), that aids specific remote areas in poverty reduction by generating net benefits, and Community Benefit Tourism Initiatives (CBTIs) that helps distribute and transfer benefits to communities through tourism initiatives without control of projects (Simpson, 2008), as well as Donor-Assisted Community-Based Tourism (DACBT; Harrison and Schipani, 2007) that alleviates community poverty by promoting subsistence economies and maximizing the use of natural resources in order to obtain benefits and create community enterprises for further income. In sum, the abovementioned works paved a line of initiatives necessary for communities to manage and control tourism planning and development due to residents participating in tourism activities in their own local communities, and offering tourism products which the tourists are seeking and help to determine the number of tourists visiting the area.

### Importance of CBT

Considering tourism as an optional tool to strengthen local economies, CBT becomes a poverty reduction tool that aids local community in various ways, such as by offering educational opportunities, environment conservation and income-generating activities (Cooperation, 2014). Hence, principles and mechanisms for developing CBT are mainstream (Bramwell and Sharman, 1999). Although CBT can increase benefits for the community and reduce negative impacts obtained from the use of community resources, CBT calls for an effective long-term plan. Hence, a tourism project should firstly perform by empowering local communities in rural areas in order to advance their potential and utilize their land and resources for community development (Mearns, 2003). Thus, with respect to this kind of tourism, possible solutions have emerged in order to deal with the negative impacts and problems of mass tourism in developing countries, consequently, to be future-oriented toward development planning for community improvement.

### Objectives of CBT

The main purpose of CBT is to include local communities in tourism by managing tourism resources and by providing further fundamental infrastructure such as accommodation, restaurants, and additional services to host tourists. Simultaneously, further elements should be added to the communities, such as healthcare, transport systems, and learning and training sites or providers (López-Guzmán et al., 2011). Following on from Hiwasaki (2006), CBT typically has four objectives. (1) Conservation of resources: sustaining the environment and bringing about positive impacts on both natural and cultural resources in the area through tourism. Consequently, tourism creates value. (2) Social and economic development: delivering a new approach to local economic development, in which costs and benefits are equitably distributed to residents participating in tourism activities. (3) Empowerment and ownership: increasing empowerment and ownership which is recognized by local communities by allowing local residents to participate in appropriate tourism planning and management. (4) Quality visitor experience: ensuring tourists partake in authentic and meaningful experiences through social and environmental responsibility.

### Previous Studies

As Table 1 illustrates, academic literature shows that many CBT projects have been introduced in Africa (Briedenhann and Wickens, 2004; Lepp, 2007; Manyara and Jones, 2007; Novelli and Gebhardt, 2007; Kibicho, 2008; Sebele, 2010), Asia (Hiwasaki, 2006; Nyaupane et al., 2006; Harrison and Schipani, 2007; Okazaki, 2008; Harris, 2009; Yang and Wall, 2009), Latin America (de Holan and Phillips, 1997; Zorn and Farthing, 2007; Trejos and Chiang, 2009), and Oceania (Dyer et al., 2003).

**TABLE 1:** Academic literature in many CBT projects in Africa, Asia, Latin America, and Oceania.

**Locations**	**Findings**
South Africa	Development opportunities of tourism projects in rural areas are acquired by formalizing participatory management among the local communities and public sectors (Briedenhann and Wickens, 2004)
Uganda	Residents have positive attitudes toward tourism, stating that it creates community development, generates income, and brings random good fortune (Lepp, 2007)
Namibia	Benefits of conservancies can only be realized by creating optimal tourism initiatives and initiating new approaches of successful tourism projects in younger generations (Novelli and Gebhardt, 2007)
Kenya	Conservation orientation of Community-Based Ecotourism (CBE), with support agencies’ approaches involving investment that promotes neocolonialism and reinforces dependency (Manyara and Jones, 2007)
Kenya	For success in CBT projects, high levels of leadership is required in order to operate their own resource in accord with the community’s benefits (Kibicho, 2008)
Botswana	Joint ventures in local communities with proper management can preserve natural resources and provide sustainable livelihood opportunities by increasing levels of participation in tourism activity (Sebele, 2010)
China	Degrees of economic leakage of a destination varies according to local involvement together with tourist number and type (Nyaupane et al., 2006)
Japan	In Japan, proper management of a park system can build tourism actors’ unity in which the mechanism becomes an optimal solution to ensure community participation (Hiwasaki, 2006)
Laos	DACBT and business operation support of tourism enterprises can be a viable tool for poverty reduction and financial availability in rural communities (Harrison and Schipani, 2007)
Philippines	CBT cannot be predicted to result in success because there are always unique factors in a particular case which may lead to different consequences. The model proposed in the work can initially evaluate the situation of tourism development in a community and suggest further projects for the community to launch. (Okazaki, 2008)
Malaysia	PPT in Bario is not only providing job opportunities to locals but also reinvigorating economic regeneration through empowering local residents and involving them in tourism activity. (Harris, 2009)
China	Diverse and high-quality ethnic products, especially locally made products, must meet domestic and international market demand in order to give tourists an authentic experience (Yang and Wall, 2009)
Cuba	When tourism activities are appropriately managed by local communities, opportunities are created to eradicate poverty and effect commodity dependence. However, there is always a threat brought to the local economy through the management (de Holan and Phillips, 1997)
Peru	The advantages and disadvantages of an outsider role in helping communities operate tourism are determined and perceived only by the communities itself. Forming alliances with the outsiders could facilitate communities’ control over resources as long as tourism practice is done in close contact with the supporters and their relationship is developed. (Zorn and Farthing, 2007)
Costa Rica	Benefits of CBT not only allow tourists to perceive inexperienced life in rural communities, but also strengthen and promote business development. As a result, CBT generates employment and local residents begin to embark on small enterprises. (Trejos and Chiang, 2009)
Australia	Cultural tourism helps communities break away from dependency, if communities can properly manage uncertainty about the exact nature of the community’s equity (Dyer et al., 2003)

Generally, local communities suffer from a lack of financial resources to support project initiatives, probably due to the level of host involvement in management and the degree of economic leakage and local control. There may be conflicts between different actors involved in tourism areas where the local government is involved (Nyaupane et al., 2006), which results in inadequate community priorities (Manyara and Jones, 2007). In this sense, CBT helps avoid the conflicts by enabling coordination between several different types of policy and creating unity for exchanging knowledge and thoughts between all members of the community (Kibicho, 2008). Finally, one sensitive controversial issue in academic literature concerns the number of tourists who visit the area. The number of tourists can vary based on travel style which depends on motivation, development of destination, event selection criteria, and involvement (Priporas et al., 2018). Nyaupane et al. (2006) added that fewer tourists is better because it allows a greater degree of interplay with valuable community resources, such as living culture and heritage, and prevents interference in private areas of the local culture from tourists. However, it is important to note that income generated by tourism with fewer visitors will be limited.

As tourists are seeking activities they are inexperienced in the locality (Valek and Fotiadis, 2018), CBT brings benefits for the communities in a variety of ways. In practice, CBT fulfills what tourists are seeking by allowing them to understand and learn about community culture and traditional lifestyles and to interact with resources of the communities. In the meantime, this event generates income for the communities and also generates tourism income for the communities. Community participation has been praised for its effective community development and is also a major key factor in successful CBT. This is emphasized by a study that showed when level of participation in an activity increases, the participants receive greater benefits generated from such an activity (Chen et al., 2020). According to Tosun (2000), community participation principles promote sustainable tourism development, provide employment opportunities for locals, and subsequently, produce greater outcomes for development taking place in the locality. In fact, unless the involved effort has reflected benefits, the community will be unlikely to participate (Murphree, 2005). This is also found in a study by Lea (1988) that showed tourism will not reflect back benefits and is less likely to generate sustainable outcomes if resident empowerment and participation fail to take into account the decision-making process of tourism development.

### Benefits and Challenges of CBT

Community-Based Tourism is an alternative optimal means for conserving natural and cultural resources and developing tourism projects. It is one of the potentially viable options to tactically sustain local livelihoods and eradicate poverty, as well as address the existing global challenges of tourism development (Timothy and Tosun, 2003). The benefits of CBT are differentiated based on current community conditions and can be seen in various perspectives such as economic profits, environmental protection, and quality of life. One of its goals is to create jobs and economic security in communities (Tamir, 2015). Jobs such as guides, travel agents, security guards, and cleaners are especially indicated in employment and even unskilled workers will be hired as CBT raises the need. Sebele (2010) stated that as long as there is waged employment in the locality, community members eradicate poverty and lift their standard of living.

Furthermore, the core of CBT is active community participation which bring about a positive impact on community resources and is considered as a development strategy that responds to community needs (Tosun, 2000). Choi and Sirakaya (2006) stated that communities can take control over their resources and manage tourism activity after the communities have accomplished tourism development. As a result, the communities obtain a greater benefit. When using tourism to call for community development, it is reliable in principle, but in practice, it faces some challenges (Scheyvens, 2002) and these must be identified in an initial stage of planning to ensure the possibility of success. A challenge can be related to the heterogeneous nature of communities or community members, for instance, class, gender, and ethical factors, which may negatively affect the intention of participation. Scheyvens (Scheyvens, 2002), identified that inadequate resources, information, and consultation to make an effective engagement with stakeholders can lead to vulnerability in tourism. Financial risks are always an initially significant factor for business success or failure in communities (Korol and Spyridou, 2020). When finance is unavailable at a local level, communities will lose their own control of resources to outside enterprises (Scheyvens, 2002). It is noted that in order to anticipate the vulnerabilities and prepare backup plans, a financial warning system is essential for business (Korol and Spyridou, 2020).

### CBT in Thailand

The interests in experiencing the traditional way of life and cultural heritage along with the interests in improving the socio-economic status of local communities, many of which are rural and socioeconomically marginalized, have led to the development and promotion of CBT. In Thailand, several local destinations offer tourism in the form of CBT and related tourism structures such as homestay and agro-tourism. For decades, CBT in Thailand has been developed to create opportunities for sustainable development and address financial shortages in communities through participation enhancement. Since then, CBT enterprises have been established in many parts of Thailand. Some destinations were reported to have successfully aided in local communities in achieving the basis of CBT, while many did not (Boonratana, 2009). Essentially, Boonratana (2009) had reported observations that a number of tourist destinations knowingly or unknowingly do not implement the ideals and principals of CBT. The same study also indicated that several destinations and product and service developments are managed by a few community members or by external businesses with little or no participation from the local communities, which results in a loss of control. In Thailand, the terms of “homestay” and “community-based ecotourism” are often used synonymously. CBT, community tourism, and ecotourism are frequently labeled as “One Tambon One Product.” Nonetheless, any form of tourism in Thailand associated with local communities is regarded as CBT and community-based ecotourism. Suansri (2003) summarized the implementation of CBT and relevant terms in Thailand as complex and confusing, and that CBT in Thailand is lacking a standardized terminology.

## Methodology

### Research Design and Methods

**FIGURE 1:**
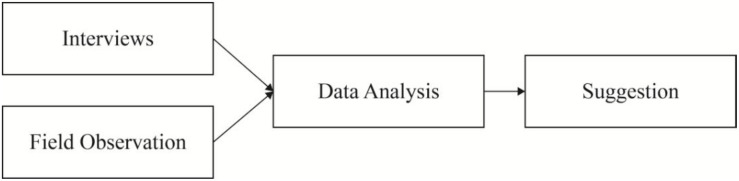
Research methodology employed in the study.

As Figure 1 illustrates, this study employed a qualitative research design, relying on field observation and interviews. As the value of qualitative research is increasingly being recognized, qualitative methodologies intend to generate knowledge grounded in human experiences (Sandelowski, 2004). These kinds of methods can offer insights to the question of why people engage in particular actions (Rosenthal, 2016) and be used when analyzing large qualitative data sets. It is imperative to conduct this analysis in a rigorous and methodical manner to obtain meaningful and useful results (Attride-Stirling, 2001).

Two types of primary data collection are administered: one focuses on a self-administered field observation taking place in the community and the another aims at interviews with community committee members and local residents. Based on methodologies in literature review, these qualitative methods are used due to methodology designs employed in previous studies employed not adequately explaining phenomenon explored in qualitative studies. In other words, it allows for identifying gaps in the literature which this study can attempt to address.

While the field observation method of data collection is used to understand how groups of people interact and behave in a particular context (Hammersley and Atkinson, 2007), interviews are used to ensure that interviewees clearly understand given questions and to ensure information accuracy. Additionally, it allows for a better understanding of context within a given scenario and shows how people interact in a specific field. More unexplored and unexpected answers can be given in individual questioning. Based on the literature, many attempts have been made in order to initiate and carry on tourism projects in specific rural communities. However, some are properly managed and accomplished. This raises a question, “What are the benefits and challenges that are derived from community resources in respect of CBT?” To answer the research question, three key sub-questions are posed: what are the challenges that are derived and distributed to the community in respect of CBT, what are the benefits that support CBT development in the community, and what is the solution to address the identified challenges? By using the research methods, the paper could contribute a deeper understanding on CBT in Thailand and further provide benefits to the relevant industries. In sum, qualitative methods provide precise answer to specific questions and reach a richness of description and explanation of nearly unidentifiable local contexts (Miles et al., 2014).

### Data Collection

As can be seen in Table 2, the methodology used for this exploration begins with informal face-to-face interviews and free discussions with community committee members and local residents. Community committee members are regularly confronted with benefits and challenges in tourism development, resource management, and inducing participation. On the other hand, local residents relate to tourism activities and hospitality industries, taking into account those who have settled in and are intimately acquainted in the area. With aspects and opinions of the community committee members and local residents, interviews are conducted to obtain and perceive the basis of community benefits and challenges, how they are derived and distributed to the community, which constraints have been encountered, and the nature of interaction and community participation. In addition, meeting dialogues are audiotaped openly for the purpose of transcription and analyses. This relies on the collaboration of the local residents in Muen Ngoen Kong community.

**TABLE 2:** Participant information, interview time duration, and field observation notes.

**Sources**	**Categories**	**Interviewee job titles**	**Interview duration (min)/no. of notes**
Interviewee A	Local	Hostel Owner	62.14
Interviewee B	Local	Hairstylist	62.38
Interviewee C	Local	Hostel Receptionist	64.40
Interviewee D	Local	Grab Driver	23.42
Interviewee E	Committee	Diner Owner	64.29
Interviewee F	Local	Housewife	19.20
Interviewee G	Committee	Grocery Store Owner	57.45
Interviewee H	Committee	Grocery Store Owner	57.45
Interviewee I	Local	Coffeehouse Owner	27.46
Interviewee J	Committee	Landlord	62.25
Field Observation Notes	Researcher	–	27

The aim of this paper is to investigate the benefits and challenges of a specific geographical area, Muen Ngoen Kong community in Chiang Mai, Thailand, and to discuss suggestions obtained from interviews in relation to existing knowledge of CBT development to address identified shortcomings found in terms of community tourism. In order to achieve these purposes, data collected from interviews were obtained after two research site visits between the middle of August in 2018 and late February in 2019 (a total of 7 months). Meeting with the community was established by the support of a lecturer in Chiang Mai Rajabhat University. Since the researcher was introduced to the community, the connection and relationship with the community was continuously sustained and the communication and opinion sharing occurred through social media platforms (LINE group and Facebook).

During August in 2018, the first visit was held and the meeting with community leaders and committees was also conducted. The researcher was given a 1-h community tour by a community committee. Geographic information, community facilities, important historical sites, culture, and living style were introduced. Informal interviews with community leader, committees, and several locals were spontaneously conducted during the meeting at Muen Ngoen Kong community office. The interview discussed major challenges that the communities had faced and solved, donor funds from the local government, and local perspectives toward tourism in the community.

However, the second visit was held in early February. Field observations was adopted and lasted from February 16th until 26th (a total of 10 days) and 27 important notes were created. The noted reports involved three main topics: benefits, challenges in relation to CBT development, and suggestions to address identified shortcomings. On the other hand, 10 interviews with community committees who are regularly confronted with benefits and challenges in tourism development, resource management, and community participation and local residents who are involved in tourism and hospitality industries, taking into account those whom have settled in and are intimately acquainted with the area, were conducted.

As can be seen in Table 2, the informant careers are diverse, ranging from a diner owner, two grocery store owners, a landlord, hostel owner, hairstylist, hostel receptionist, grab driver, housewife, and coffeehouse owner, four of whom were community committees. Before interviews, the purpose of the interview and the theme created by the researcher which was to be discussed (generally the same) was informed to and clarified with the interviewee. Informants were asked and gave permission to be interviewed and audiotaped openly for the purpose of transcription and analyses. The interviews were mostly conducted at the interviewee’s home or workplace where they could feel comfortable cooperating with the researcher. The interviews lasted between 30 min and 1 h and the interviewees preferred to speak in the Thai language as it is the common language spoken in daily life. Total interview duration was 7 h 25 min and a 204-page conversation in Thai was obtained from the interviews and transcribed and translated into English.

### Data Analysis

This research uses Braun and Clarke’s (2006) thematic analysis procedure, a six-phased method, to analyze raw data and convert it into useful results. To establish and fulfill the trustworthiness assessment, as it is qualitative data, Lincoln and Guba (1985) outline criteria for trustworthiness during each phase of the thematic analysis. Indeed, Braun and Clarke’s (2006) six-phased procedure is iterative and reflective as it has been developed over time and links a continual moving between phases. This particular procedure includes revisiting collected data, generating codes, establishing themes, re-examining themes, defining themes, scripting, and, finally, reporting findings. Relying on the literature review, interviews, and field observation notes, suggestions are given in order to address the identified shortcomings.

### Description of Research Site

**FIGURE 2:**
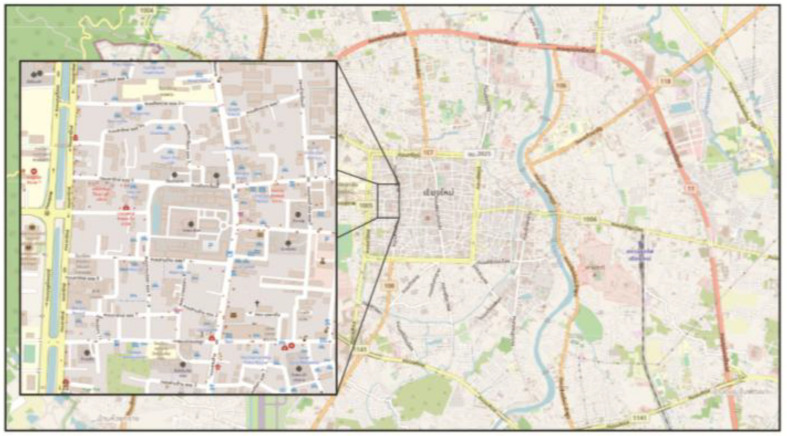
Location of Muen Ngoen Kong Community in Chiang Mai, Thailand.

Muen Ngoen Kong community, which is situated in the largest city in northern Thailand, Chiang Mai, has occasionally been overlooked, and has not yet developed structured tourism as more emphasis is placed on other nearby communities. Set up in 1999, Muen Ngoen Kong is a community which was established by the collaboration of local aboriginal peoples. The purposes of the establishment are to solve problems that occur occasionally, to develop livelihoods, and to manage common property resources in order to present opportunities for economic development through the sustainable use of existing cultural resources, especially historical sites. It has an approximate area of 2.4 km^2^. Compared to other communities in Chiang Mai, the community, as shown in Figure 2, is rather small. Arranged in the shape of an irregular hexagon, the community is situated in the Chiang Mai Old City, bordered by Khuan Khama community on the north, Buak Hard community on the south, Samlaan Road on the east, and Arak Road on the west, some 700 km from Bangkok, the capital city of Thailand. Chiang Mai Old City is one of the main places of interests for tourists. In the past, Chiang Mai Old City was founded by the first king of Lanna, King Mungrai, 720 years ago and the city used to be the center of the Lanna Kingdom which was a melting pot of culture and crafts. With the legacy of Lanna’s history, Lanna and Chiang Mai became the center for historical sites to visit in Asia.

Muen Ngoen Kong community has a population of 1882 people (844 men, 1038 women, and 773 households in the 2018 census). The community is governed by a group of eligible and elected people including a leader, a vice leader, and seven committee members who were voted by the community citizens. In this community, tourism development has not been advocated for and prioritized yet as well as in other nearby communities. As a matter of fact, there are currently a few hotels and hostels in the hands of multinational foreign and local owners. With the respect of cultural resources in the community of Muen Ngoen Kong, and more particularly ancient historical sites, such as Phra Singh Temple and Prasat Temple, cultural preservation is the most important factor to be considered. Financial contributions made by the government have been very crucial in this area, creating and improving small businesses managed by local peoples. Furthermore, the attempt to increase tourist volume through the role of cultural resources has been noted as significant as well.

## Results

### Research Results

**FIGURE 3:**
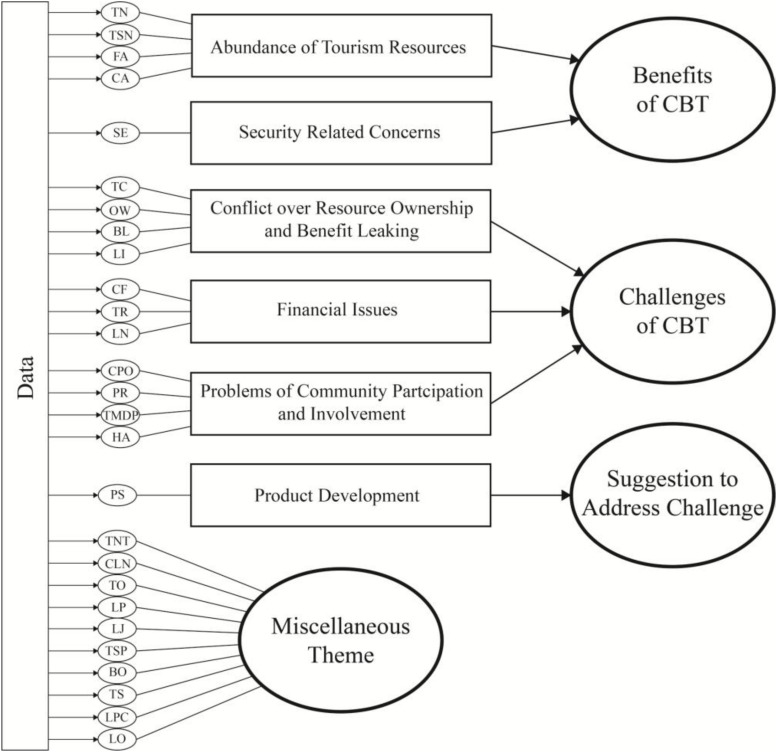
Thematic network.

To determine the understanding of participants, thematic analysis were employed and applied to analyze all the transcripts, and three key concepts were derived from the process. Based on research questions, these categories have been labeled as “Benefits,” “Challenges,” “Suggestions,” and “Miscellaneous Theme.” Miscellaneous Theme was conducted to store irrelevant notes that did not belong to any category. Unavoidably, participants’ understanding may overlap with existing categories or each other. The results consider that understanding of a specific context are well interpreted and concepts are relative to each other. As Figure 3 illustrated, interviews and field observation highlighted three key themes in Muen Ngoen Kong community and proved that the community has difficulty in participating in tourism management and planning. These include Challenges, Benefits, and Suggestions.

### The Challenges of CBT

Muen Ngoen Kong community has several challenges toward CBT, these include the following:

#### Conflict Over Resource Ownership (Tamir, 2015) and Benefit Leaking

An important determinant for success and failure of CBT development is ownership issue (Denman, 2001). When tourism reaches a sufficient level, communities are involved in the industry. The analysis indicates that local residents lost a number of valuable natural resources, located in Muen Ngoen Kong community; the most important of these is land to outside investors. It is stated “a large number of guest houses are owned by outsider investors (Interviewee G).” It is indicated that to be successful in tourism management, resources must be managed efficiently and collectively, community member should be resourceful (Fotiadis et al., 2019). In this regard, the community determined that loss of the right to possess and own the land has incurred more costs than benefits. An interviewee added “locals might not perceive its importance (of land ownership), but others do. That’s why they keep stepping into this area (Interviewee J).” In the perspective of a community committee, the resources ownership issue is considered as a major challenge. Several major service businesses operating in the community are owned and managed by outside investors who have signed long-term contracts (over five years in duration) with local landlords. A community committee described the impact of having outsiders in the community: Due to the number of non-registered populations and ownership by the outside investors, the community committee members had indicated that landlords have doubtlessly lost rights to possess and control land resources. From this an insight into the negative responses to leadership from the community hosts and local entrepreneurs was gleaned, as it is perceived that they cannot deal with the available resources productively (Fotiadis et al., 2019).

#### Financial Issues

Almost every informant in this study revealed that financial resources and capital issues remain a big challenge for them to set up facilities in line with the minimum requirements. Developing tourism facilities in communities frequently requires funding or donor support from government sectors or stakeholders (Reed, 1997). Hence, ownership issues, community interest, and knowledge are not the only key factors for community members to be involved in CBT, but also available financial resources at a local level. The need for financial resources for tourism investment is inadequate and not readily available in most cases, especially in developing countries (Long, 1991; Pearce, 1991; Tosun, 1998) and also in the community. A local stated “as I noticed, if the resource is adequate, local government should provide a steady and progressive development, right? (Interviewee B).” This has become as a major challenge to implement tourism development in Muen Ngoen Kong community. Finance for tourism is insufficient at a local level and is derived from donors and a small number of government sectors. However, a community that is managing to survive with the limited and inadequate financial support from the government has shown high levels of effective cooperation in order to deal with the shortage that occasionally occurs (Fotiadis et al., 2019). It is mentioned “in fact, locals are short of money to run a business and mostly, the outsider investors have a larger capital (Interviewee E).” With respect to non-local capital, the possibility to completely control and manage tends to be difficult and becomes an obstacle of the community to improve wellbeing and encourage a participatory approach.

#### Problems of Community Participation and Involvement

Community-Based Tourism involves local participation, with the handing over of control to communities resulting in more benefits to livelihood (Mitchell and Reid, 2001). While CBT offers the high possibility to create jobs and increase a community’s income-generating capability through tourism development, local participation provides community members a greater opportunity to obtain benefits from the development (Tosun, 2006). Since local residents are part of the tourist product, the success of tourism is decided by the participation and cooperation of the local community.

From the analysis, it is explicit that community participation and involvement are a vital part of shaping and achieving the expected developments in the community. A community committee stated that they organize some mutual activities for building intimacy through friendship among residents, for example, annual activities and festivals, maintenance of tourism historical sites and heritages, and street cleaning. A committee said “when we have street cleaning or maintenance of tourist sites, we prepare all the equipment and start doing it together (Interviewee E).” The same committee added “we informed business owners [of] the cleaning schedule and they sometimes gave us packs of bottled water.” However, it is more likely to be unsuccessful in different groups of local people. Despite the fact that the committee members regularly hold meetings at least once a month to follow up on local’s complaint letters and give written reports to community representative members, the closeness between local residents and the committee members and people’s attitudes to tourism development in the community is not likely to be improved. This reduces the possibility of board members to realize and understand local aspects and demands toward tourism development.

Essentially, the results of an interview revealed that some community members are participating in activities which took place in the community and was organized by tourism offices in regard to CBT development in the area. However, there was not much cooperation while the activities ran their course. A local argued to the above committee’s words “locals attended the activities in the temple (a common place that locals gather) but showed less cooperation (Interviewee B).” Thus, this ensures that the participation at the CBT development activities was restricted by receiving less cooperation with activities in terms of tourism development.

### The Benefits of CBT

Muen Ngoen Kong community revealed the advantages it gained toward tourism resources and improvements to community security; these include the following:

#### Abundance of Tourism Resources

Muen Ngoen Kong community is promoted as a site for community tourism in Chiang Mai where famous ancient architecture, community culture, history, and a unique slow-paced way of life are major tourism attractions. The community is south-west of Chiang Mai city, which is a quiet quarter. Tucked away from the tourist-flooded streets and popular grand department stores, tourists will be surprised by the living city spirit. Local Lanna temples, traditional craftsmanship, edgy graffiti, Thai cooking classes, and spicy street food are the best way to understand their true way of life.

According to the tourist’s opinions, the purpose for visiting Muen Ngoen Kong community is to witness a perfect living city spirit, traditional workshops where they make clothing by hands, contemporary street art, and traditional food. The tourism products are the purely tangible cultural heritage and a way to interact with the local community. On the other hand, locals indicated that tourists visited their community with the aim to experience the community’s cultural attractiveness. Based on the analysis and field observation notes, potential attractive tourist destinations in the community are as follows:

• Wat Phra Singh
  The second most reputable active temple in Chiang Mai after Wat Phra That Doi Suthep is a long-established temple in which its name is literally translated as “The Monastery of the Lion Buddha.” Wat Phra Singh is very busy with visitors and worshipers all year round and is usually overcrowded during the Thai New Year festival in mid-April, and religious holidays. Every Thai New Year festival (Songkran), Lion Buddha will be presented to Chiang Mai locals in the ceremonial event around main roads for ritual baths. Through providing a cultural environment and hosting religious holiday events, visitors are aroused and the community receives attention.
• Wat Prasat
  Located opposite Wat Phra Singh in the old city wall of Chiang Mai, the founding date is unknown but is generally believed to date back to the end of the 16th century. This is an attractive temple complex and the temple contains a ubosot (ordination hall), a viharn, (assembly hall), and two chedis (pagodas). The ordination hall, assembly halls, and one pagoda are constructed in a straight line. Another chedi, a tall cone-shaped structure which enshrine important relics, flanks the west wall of each of the buildings.
  The remarkable and astonishing site is notable mostly for its viharn, whose walls are partly decorated with murals from the early 19th Century. The panels on the entrance and four sets of pillars made of teak are intricately decorated with Lanna-style carved flower motifs and animal figures of which the gilt has faded. A unique feature of this temple is the short tunnel that leads to the chedi directly behind it. While most temples usually have a set principle Buddha image in a viharn, the Wat Prasat Buddha image is in a chedi which can partly be seen from the viharn. Also, on both sides of the tunnel entrance are several seated Buddha images. One of the images is a bronze dating back to 1590, the others are stucco and of a more recent date. The temple is under the care of the Thai Fine Arts Department.
• Location
  Location is an important factor for CBT projects because the area where tourism is taking place needs to be accessible. In most cities, the best attraction is located in center, so if this is what tourists intend for their trip, tourists might want to stay in a hotel with easy access to those amenities. In this case, Muen Ngoen Kong area, where Wat Phra Singh and Wat Prasat are located close to each other, is in the western part of the old city wall of Chiang Mai, and on the main street. The surrounding area of the community is also crucial. Interesting environments and varied infrastructure enabling attractive leisure activities can be essential factors attracting business clients. The community location is considered to be an advantage because tourists can visit one or more sites all together once they arrive at the community. Furthermore, there are night markets, traditional restaurants, and street food to attract tourists other than cultural attractions.

#### Security Related Concerns

Security issues have been identified as one of the major challenges in the implementation of CBT in local communities as criminal activity and traffic incidents are a primary concern to the tourism industry (Tamir, 2015). The existence of drugs and events of theft, robbery, and begging in a destination can be perceived as harmful events to tourist flow. Committee members agreed that traffic accident issues around Muen Ngoen Kong community have been considered as the main barrier to promote tourism. Yet, traffic accidents and crime activity do not happen frequently. It is assured to be “Rarely seen. We have not been informed. Previously, it [bag snatching] happened in a small street, but it does not occur currently (Interviewee G).” However, the public may expect police to respond to these issues by focusing on its causes.

To address the issues, local governments have succeeded in strengthening and supplying effective crime prevention solutions through coordination and sharing of the best volunteer community police training programs. A committee added “we have volunteer community policemen. They are looking after us. They help monitor at night. One of our community committee [members] earned a place in the police team (Interviewee H).” Ample practice in volunteer community police roles in preventing crime have been implemented among community committee members and it has shown to reduce violence, including shootings and homicides, in neighborhoods.

In essence, community policing and its actions enable formalizing police-community collaboration and connection in which all civilians devote themselves to crime prevention through communication. However, educating the residents on security and safety and reporting crime to authorities may be required. Indeed, according to the analysis, the majority of informants reported the absence of a security problem. Seven out of 10 informants claimed that the possibility of crime occurrence in the community is very rare and it is not directed to foreign tourists. A committee added there is a higher possibility of crime happening during the festive season, and Muen Ngoen Kong community may be targeted. Thus, tourist security should always be considered and primarily prioritized in tourism planning and CBT implementation.

### The Suggestion to Address the Identified Challenges

Muen Ngoen Kong community suggested product development as a solution to address the identified challenge.

#### Product Development

In most cases, CBT is progressed and developed in accordance with community resources, CBT aims, and specific local needs due to the core of the CBT plan being to determine how best to use resources as a development tool (Scheyvens, 2002; Asker et al., 2010). However, there are many CBT projects that have failed because of the impropriety of products. The existing issues of products are that products are not designed to be demanded by buyers, and not developed in close consultation with partners who can provide training programs, necessary equipment, and market access, and without appropriate knowledge of product development, usually established by private sectors.

In the case of Muen Ngoen Kong community, informants indicated similar issues and suggested the need to increase the number of leading restaurants and develop superior products owned by the community. However, the first activity before going forward with what informants suggested above is to conduct market analysis and research for the strength and weakness of the community toward product development or business opportunities. Political situation, tourist volume, community readiness to take action to manage and tackle an incident, transport, and additional services should be all analyzed.

## Discussion

This study attempted to examine benefits and challenges of CBT in Muen Ngoen Kong community as well as suggestions in relation to local perspectives in order to address those shortcomings. This discussion addresses its current circumstances from the perspective of both community representatives and locals. This case study extends the literature by focusing on Muen Ngoen Kong community, which is located between well-known tourist destinations in the central of Chiang Mai Old City, Thailand. However, the community has been under-researched in regards to investigating CBT benefits and challenges.

### Research Question 1: What Are the Challenges That Are Derived and Distributed to the Community in Respect of CBT?

Cole (2006) pointed to the lack of ownership as a challenge to CBT, and this has been evidenced by this study. The research findings from interviews indicated the inexistence of a sense of ownership over tourism development in the community. Community sustainability is often derived from opportunities and resources to develop on their own. As land is considered as a community resource, land ownership issues become a problem for developing accommodations in the area, resulting in difficulties for CBT development. When the resource is directly related to the community’s tourism and it will become an important determinant for the community’s economy (Tamir, 2015), it becomes an issue of how the community land should be used by either the community or by foreign people.

Based on field observation notes, major service businesses are not only owned by foreign people but also by the community resident themselves. Some indigenous businesses that start their own enterprises are affected by public doubt about whether they can succeed or not. Hence, conflict over community land is sometimes raised by community capacity and intention where the community guesthouse, hostel, hotel, and homestay were constructed for business purposes. However, it is argued that tourism entrepreneurs can lead to successful rural development, if cooperation among the entrepreneurs and local government is formed (Fotiadis et al., 2019). Alternatively, handing over of land ownership to foreign people who have larger capital for investment may be a conscious attempt to foster greater community economics. But, there is a possibility that this move instead results in a benefit leaking. Surrendering control of land ownership to outsiders without building a sufficient capacity, appropriate structures, and systems for their own community is a recipe for failure.

Scheyvens (2002) also identified insufficient financial resources as another challenge to develop communities using CBT principles. The same author (Scheyvens, 2002) described that if financial resources are unavailable at a local level, there is a high possibility for communities to lose ownership of the community’s property in various ways to outsiders. From the interviews, local residents welcome and support outside donor projects taking place in the community and show an interest in tourism planning. Nonetheless, they need to accept that existing donor projects from outside will deny the community a participatory and decision-making approach. As seen in other cases, a long-term partnership between outsider stakeholders and communities can aid the communities in reaping benefits from tourism development. In this case, the community’s interest is the first concern and creating programs tailored to the need may help them the most. However, the community is in need of expertise and tools to make the programs possible. Therefore, it necessitates requesting outsider consultation. The community relies on outsider advice and know-how until they have the capability to forsake the help of outsiders, as observed in Bolivia (Jamal and Stronza, 2009).

While CBT offers the high possibility of creating jobs and increases a community’s income-generating capability through tourism development, local participation provides community members with a greater opportunity to obtain benefits from the development.

As CBT is an alternative means to enhance a significant level of local participation by allowing communities to control tourism projects, there are also other means to present wider opportunities for a participatory approach. In bicycle tourism, it is reviewed that the behavioral intentions of participants can be enhanced by having compassion, encouragement, and support of friends and family (Lin et al., 2020). Additionally, a study indicates that if a person put effort into an activity, a stronger feeling of involvement will be produced (Chen et al., 2020). However, in this case, the interviews and field observation had shown that the absence of local participation is rather obvious. Despite the fact that the intention of participants cannot be created productively in different groups of local people, it also diminished the closeness of their friendship. Significantly, with the situation of elected committee members’ minority voice, the possibility of participating in community decision-making process for cultural managements is decreased. In developing tourism communities where group cohesion is required to acquire decision-making advantages and integrating tourism, group solidarity is essential (Mitchell and Eagles, 2001; Mitchell and Reid, 2001). Therefore, management decisions should be done in close alignment with community members, because they are the ones that have a better understanding of the situation and the ones who receive the benefits that results from the management decisions (Thaman et al., 2016). It is argued that, because management in the tourism industry field sometimes requires immediate decisions (Murphy, 1985), it is difficult to ensure that all voices are represented in the decision-making process (Nault and Stapleton, 2011). However, using forecasting models can address and ease strategic and urgent decision-making because it can assess weakness in order to minimize any risks (Korol and Spyridou, 2020).

### Research Question 2: What Are Benefits That Support CBT Development in the Community?

Although tourism in Muen Ngoen Kong community revealed various challenges, the community still has strong points to support CBT development. As the interview and field observation findings indicated, tourism resources in the community serve its purpose for CBT as they are located in the community and attract tourists all year around. Tamir (2015) described that locational advantages of an area being close to a top tourist destination is presented as an opportunity for CBT development. It may be convenient that tourists can enjoy one or more destinations and CBT sites at the same time.

Although the culture of the community appreciates and attracts a number of guests, this study has shown a high level of security protection through community policing and actions. Arguably, Tamir (2015) indicated that the security issue is one of the major challenges in implementing CBT, as criminal activity threatens the tourism industry. Although the community crime scale is small, criminal activities such as bag snatches and theft are major security-related challenges toward CBT initiatives.

### Research Question 3: What Is Solution to Address Identified Challenges?

CBT projects often suffer from a lack of financial sustainability. Mostly, it is due to the absence of business expertise, knowledge among members (Forstner, 2004; Gascón, 2013; Kontogeorgopoulos et al., 2014), and access to the market. When CBT is developed based on community assets, the aim to develop a greater economy is the first major concern. Marketing notable community products and introducing special offers may become a focus for the supply side of the spectrum, but what tourists are going to buy might be overlooked. In this sense, products should be designed based on market demand. From interviews and field observations, findings mentioned the need for superior products locally owned by the community as a solution toward financial sustainability. Häusler (2005) suggested communities must conduct a market analysis in order to explore their strengths and weaknesses and then determine business opportunities. Additionally, products should be developed through a partnership in which they can assist in providing necessary skills and market access (Dodds et al., 2018), and marketing strategy can be more successful if a communication plan is built based on tourist motivation and involvement (Priporas et al., 2018). Kontogeorgopoulos et al. (2014) advised the third government agency to assist with the One Tambon One Product (OTOP) program. Because the purpose of the program is to promote the best local products at a local level, communities can adopt this program to put themselves into a proper market in order to foster community wellbeing. In the work of Kontogeorgopoulos et al. (2014), it is demonstrated that OTOP program application has assisted Mae Kampong village in developing community-based homestay projects. As a result, the village received its recognition for OTOP Village Champion in 2014.

## Conclusion

Through the case study at Muen Ngoen Kong community, Chiang Mai, Thailand, this study attempted to investigate the benefits and challenges of CBT in Muen Ngoen Kong community and discuss the suggestions obtained from two qualitative data collection methodologies: interviews and field observations, and the thematic analysis process. It concluded that CBT efforts in the community affect the livelihoods of local residents in two different ways: abundance of tourism resources and security-related concerns. Community-Based Tourism contributed its benefits to community subsistence by allowing large volumes of tourists seeking a new approach to discovering cultural destinations and experiencing local culture in order to involve themselves in their travels, and providing criminal protection programs to ensure tourists’ and local’s safety. In most cases, the security issue is a major challenge that hinders the progression of tourist development. However, in this case, the community succeeded in strengthening and supplying effective crime prevention solutions through coordination with local government.

Despite the attractiveness of tourism resources and the benefits of a volunteer police program in addressing security issues, there have been several barriers to sustain the community in the long-term. The key challenges that determine the success of CBT are: conflict over land ownership and benefit leaking, financial issues, and problems of community participation and involvement. The community shared some of these challenges which may occur in many developing countries. Several studies had indicated that, without creating opportunities for local residents to possess their land and to participate in the decision-making process, benefits that are generated from the tourism development are nearly impossible to be sufficient (Long, 1991; Tosun, 1998; Clancy, 1999; Timothy, 1999). According to this challenge, it was revealed that some community members felt the profit from tourism often does not filter down to the local economy and the costs they incurred far outweigh the benefits. Additionally, it may become a critical issue that locally owned small businesses are operating against stronger competitors and under imperfect market conditions. Consequently, they lost the essentials for their wellbeing.

CBT initiatives in relation to product development must be developed according to the strength of the community and should embrace collaborating with third government agencies. While many CBT initiatives emphasize the importance of collective management in communities, external tourism enterprises with the potential to provide knowledge, market access, and additional sources are often overlooked (Dodds et al., 2018).

## Suggestion for Future Studies

The research findings of this study indicate that several challenges have had a negative impact on the development of CBT at the research site. Additionally, this study investigated benefits and opportunities that should be strengthened and developed in the future steps of community development. Based on the findings of the research, solutions to address the identified challenges were suggested by local residents. However, product development as the solution to overcome several challenges remains an issue of how community land should be used by the community or foreign people.

Essentially, to increase the consistency of coding procedures, multiple coders or a team of researchers with expertise in qualitative research and coder comparison analysis should be applied. This quality assurance method will ensure that the coding process and interpretation are illustrative of the data. If multiple coders or coder comparison are not possible, detailed notes of any decision that had been made should be kept.

In qualitative research, the researcher is a part of the research process itself. Knowing biases occur during the interpretation and analysis processes should be reported openly in manuscripts. Hence, it must be clear to the reader on how the background could produce biased findings.

With the rich conversation transcribed from the recordings, there should be hidden and unexplored challenges, benefits, and solutions regarding the possibilities of CBT development. Careful coding processes and interpretations with multiple coders should be reprocessed in order to discover hidden challenges and benefits as well as solutions in relation to CBT. Another limitation that should be considered is the generalizing of findings due to unique cultures and locations. Nonetheless, it can be the basis for more consideration of community development contexts in coping with CBT.

## Data Availability Statement

The raw data supporting the conclusions of this article will be made available by the authors, without undue reservation.

## Ethics Statement

Ethical review and approval was not required for the study on human participants in accordance with the local legislation and institutional requirements. The patients/participants provided their written informed consent to participate in this study.

## Author Contributions

Y-CL made substantial contributions in exploring research concept, developing the research framework, and designing the methodology. PJ made substantial contributions in data collection, writing the manuscript, and searching for references. Both authors made substantial contributions in data analysis, result interpreting and discussing, drafting the manuscript, and writing the authors’ response notes.

## Conflict of Interest

The authors declare that the research was conducted in the absence of any commercial or financial relationships that could be construed as a potential conflict of interest.
